# In Vivo Dynamic Coronary Arteries Blood Flow Imaging Based on Multi‐Cycle Phase Clustering Ultrafast Ultrasound

**DOI:** 10.1002/advs.202505485

**Published:** 2025-06-30

**Authors:** Hao Yu, Jiabin Zhang, Feng Feng, Jinyu Yang, Yu Xia, Yunlong Zhao, Jue Zhang

**Affiliations:** ^1^ College Of Engineering Peking University Beijing 100871 China; ^2^ College of Future Technology Peking University Beijing 100871 China; ^3^ New Cornerstone Science Laboratory CAS Key Laboratory of Biomedical Effects of Nanomaterials and Nanosafety and CAS Center for Excellence in Nanoscience National Center for Nanoscience and Technology of China Beijing 100190 China; ^4^ Academy for Advanced Interdisciplinary Studies Peking University Beijing 100871 China

**Keywords:** dynamic coronary arteries blood flow imaging, full cardiac cycle, multi‐cycle phase clustering, super‐resolution ultrasound

## Abstract

Echocardiography is the most primary clinical modality for dynamic cardiac imaging. However, the vigorous motion of myocardial tissue inherently causes inevitable signal aliasing between blood flow and myocardial tissue in traditional Doppler ultrasound imaging, constituting a persistent challenge in echocardiography for decades. Particularly for low‐velocity flows like coronary artery blood flow, the Doppler frequency shifts completely overlap with myocardial tissue signal shifts, rendering them virtually undetectable through conventional echocardiography. This study pioneers a paradigm shift by abandoning traditional Doppler‐based blood flow imaging, and develops a novel multi‐cycle phase clustering method for tissue clutter suppression, successfully extracting dynamic cardiac blood flow signals with clear visualization of both coronary artery flow and cardiac chamber flow throughout the full cardiac cycle. Furthermore, through integration with super‐resolution ultrasound reconstruction techniques, enhanced spatial resolution in cardiac blood flow imaging is achieved. This breakthrough establishes a novel in vivo cardiovascular flow imaging approach characterized by high spatio‐temporal resolution, safety, and clinical accessibility, demonstrating significant potential for advancing the diagnosis of cardiovascular diseases.

## Graphical abstract

This study addresses a critical gap in ultrafast ultrasound cardiovascular flow imaging and introduces a transformative approach, Clustering Singular Value Decomposition (cSVD), which enables dynamic cardiovascular flow imaging throughout full cardiac cycle without motion correction and hyper‐fast imaging strategies. It is believed that cSVD opens new possibilities for advancing the diagnosis and research of coronary artery disease.

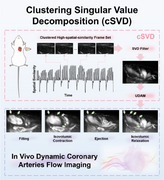

## 1 Introduction

Cardiovascular diseases (CVDs) are the leading cause of mortality globally, which accounted for 20.5 million deaths in 2021, comprising approximately one‐third of all global deaths.^[^ ^1^ ^]^ Most cardiovascular diseases are caused by coronary artery lesions, making non‐invasive cardiovascular imaging, particularly coronary artery imaging, extremely important.^[^ ^3^, ^4^ ^]^ At the same time, it remains one of the major challenges in cardiovascular disease research.^[^ ^5^ ^]^

Current clinical modalities for in vivo cardiac imaging primarily include echocardiography, cardiovascular magnetic resonance imaging (CMRI), cardiac computed tomography angiography (CCTA), and digital subtraction angiography (DSA).^[^ ^6^, ^7^, ^8^, ^9^, ^10^, ^11^, ^12^, ^13^, ^14^, ^15^, ^16^, ^17^, ^18^, ^19^ ^]^ Echocardiography remains the most critical clinical imaging modality for cardiac assessment, but traditional echocardiography is permitting only gross evaluation of cardiac structural and intracardiac blood flow while failing to directly visualize coronary arteries.^[^ ^6^, ^7^, ^8^, ^9^ ^]^

With recent advances in ultrafast ultrasound technology,^[^ ^20^, ^21^, ^22^, ^23^ ^]^ ultrasound imaging enables visualization of coronary blood flow during diastole.^[^ ^5^, ^24^, ^25^, ^26^ ^]^ Recently, Demeulenaere et al.^[^ ^24^ ^]^ and Yan et al.^[^ ^26^ ^]^ proposed a series of singular value decomposition (SVD) clutter filter‐based motion correction strategies, which have been applied to ultrasound cardiovascular flow imaging, offering effective imaging solutions for cardiovascular researches.^[^ ^24^, ^27^, ^28^ ^]^ However, the current implementations are restricted to quasi‐static imaging windows and imaging frame rate, thereby missing critical physiological information during peak systole and rapid filling phases where coronary flow variability is most pronounced,^[^ ^24^, ^29^ ^]^ and incapable of capturing dynamic cardiovascular flow imaging throughout the full cardiac cycle, leaving a significant gap in diagnostic capabilities. Besides, these motion correction methods often become ineffective when the region of interest (ROI) of imaging target undergoes significant deformation or even moves outside the imaging range, which is unavoidable in 2D ultrasound imaging.^[^ ^24^, ^27^ ^]^ In addition, hyper‐fast ultrasound based cardiovascular flow imaging^[^ ^5^, ^24^ ^]^ with 2000 or higher frame rate brings pressure to computing and storage cost. For human transthoracic cardiovascular flow imaging, prolonged ultrasound echo time results in a decreased frame rate, which makes it hard to meet the stringent frame rate requirements of hyper‐fast imaging strategies, thereby limiting such methods.

While alternative imaging modalities cannot match the temporal resolution of ultrafast ultrasound, they still can achieve cardiac imaging through exploitation of their inherent physical imaging properties and synchronization with cardiac cycle periodicity. Notably, CMRI uses electrocardiography (ECG) and respiratory gating to combine images from the same phase of different cardiac cycles for image enhancement, alongside motion correction methods to improve image quality.^[^ ^27^, ^30^, ^31^, ^32^, ^33^, ^34^, ^35^ ^]^

Therefore, we considered whether we could effectively utilize multi‐cardiac cycle information to improve the sensitivity of blood flow imaging without complex motion correction and hyper‐fast imaging. Through reviewing MRI,^[^ ^27^, ^30^, ^31^, ^32^, ^33^, ^34^ ^]^ we realized that the acquired data within the same phase using ECG and respiratory gating have high‐similarity, and it is exactly the spatio‐temporal information brought by these high‐similarity frames that fundamentally enables to suppress motion artifacts. Inspired by this, we propose the Clustering Singular Value Decomposition (cSVD) from the novel perspective of spatial‐similarity clustering, which we use the inherent motion information of B‐mode ultrasound imaging and inter‐frame spatial‐similarity measurement, clustering high‐spatial‐similarity frames of the selected frame from the phase of multi‐cycle for SVD clutter filtering. We assume that its feasibility lies in the fact that, within each high‐spatial‐similarity frame sets, motive organ remains quasi‐static, satisfying the prerequisites of SVD clutter filter, which enables the suppression of motion artifacts using the internal spatio‐temporal information provided by the high‐similarity frames. Furthermore, blood flow images obtained by cSVD can be combined with the super‐resolution ultrasound reconstruction strategy^[^ ^36^ ^]^ to achieve high spatial resolution dynamic coronary arteries blood flow imaging of full cardiac cycle, opens new possibilities for advancing the diagnosis and research of coronary artery disease.

To validate the effectiveness of the proposed cSVD method, we compared the imaging quality of the traditional SVD‐based sliding spatiotemporal analysis^[^ ^25^ ^]^ with cSVD and demonstrated the interpretability of parameter setting in contrast‐free cardiovascular flow imaging. Besides, we attempted to achieve high spatial resolution dynamic cardiovascular flow imaging enhanced by ultrasound diffraction attenuation microscopy (UDAM)^[^ ^36^ ^]^of full cardiac cycle. During this process, we focus on analyzing the cSVD performance of coronary artery imaging. Additionally, multi‐slice 3D ultrasound scan was applied on whole newborn rat hearts, enabling to capture the blood flow fluctuations throughout full cardiac cycle and the corresponding cardiovascular distribution at various cardiac phases. To validate the generalizability of the proposed cSVD method across different motion patterns, we achieved contrast‐free hepatic imaging and super‐resolution ultrasound (SRUS) renal imaging with enhanced microvascular sensitivity.

## 2 Results

### 2.1 In Vivo Contrast‐free Coronary Arteries Blood Flow Imaging

**Figure 1:**
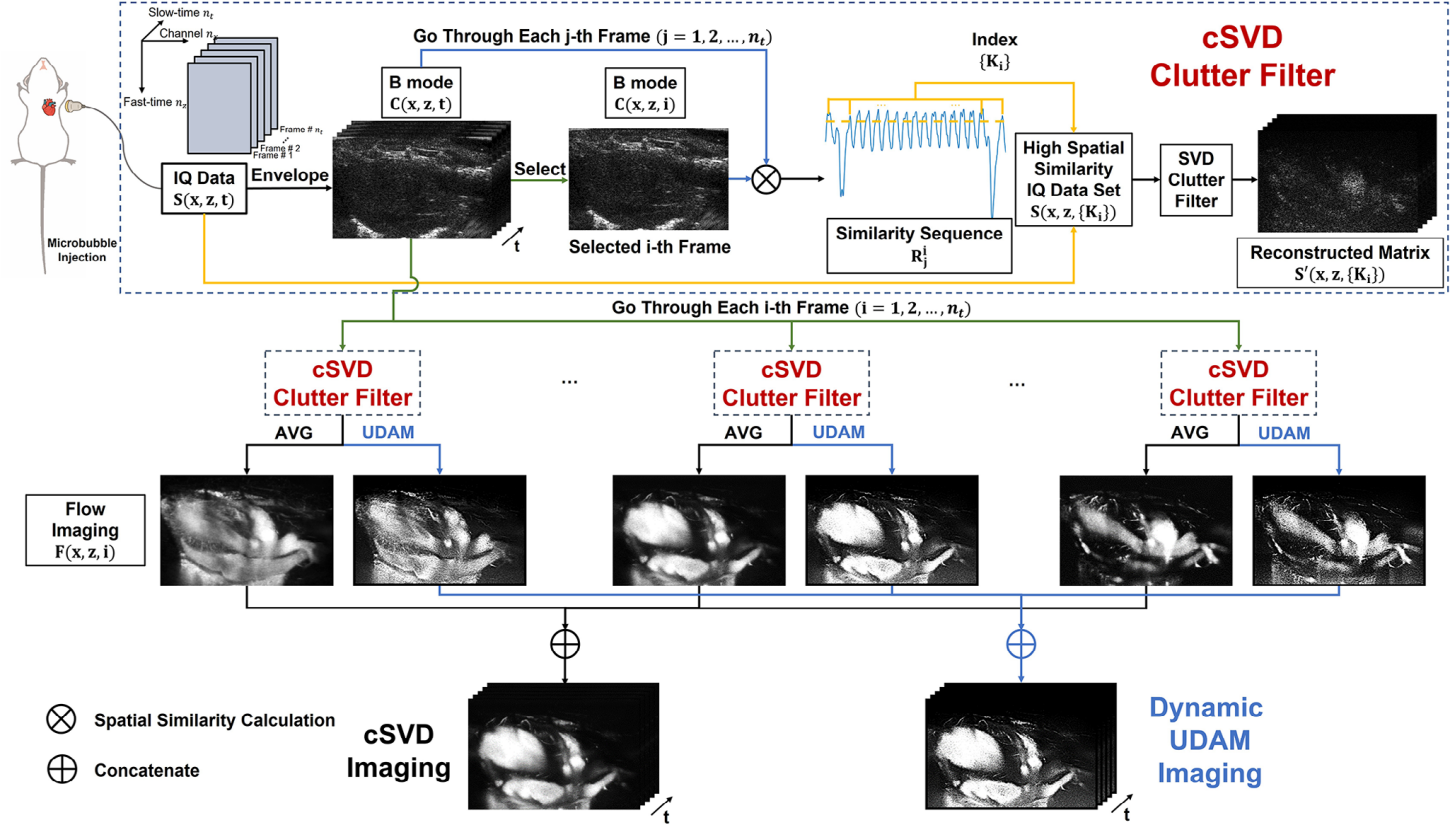
The overview of our framework. The RF data of *n*_*t*_ frames were acquired to obtain IQ data, and the magnitude of the IQ data was computed to derive B‐mode imaging results. Then, the spatial‐similarity sequence between the selected frame and all the frames was evaluated, and the top‐*M* highest‐similarity frames were selected to form a quasi‐static set, which feed into the adaptive SVD clutter filtering and reconstructed to flow imaging result via intensity average or UDAM. By processing each frame in a full cardiac cycle, the dynamic flow imaging result was obtained.

The main methodological framework of this study is shown in **Figure** **1**. To validate the cardiovascular flow imaging capability of cSVD, particularly coronary and intracardiac flow, we applied cSVD to contrast‐free imaging on free‐breathing mice. Notably, previous studies^[^ ^25^, ^26^ ^]^ have primarily focused on imaging during the diastole when cardiac deformation is minimal. However, for ultrasound imaging, at the end of ejection and isovolumetric relaxation, the intracardiac blood volume reaches its lowest level, reducing acoustic signal occlusion caused by intracardiac flow, thereby, making the coronary arteries easier to image.

**Figure 2:**
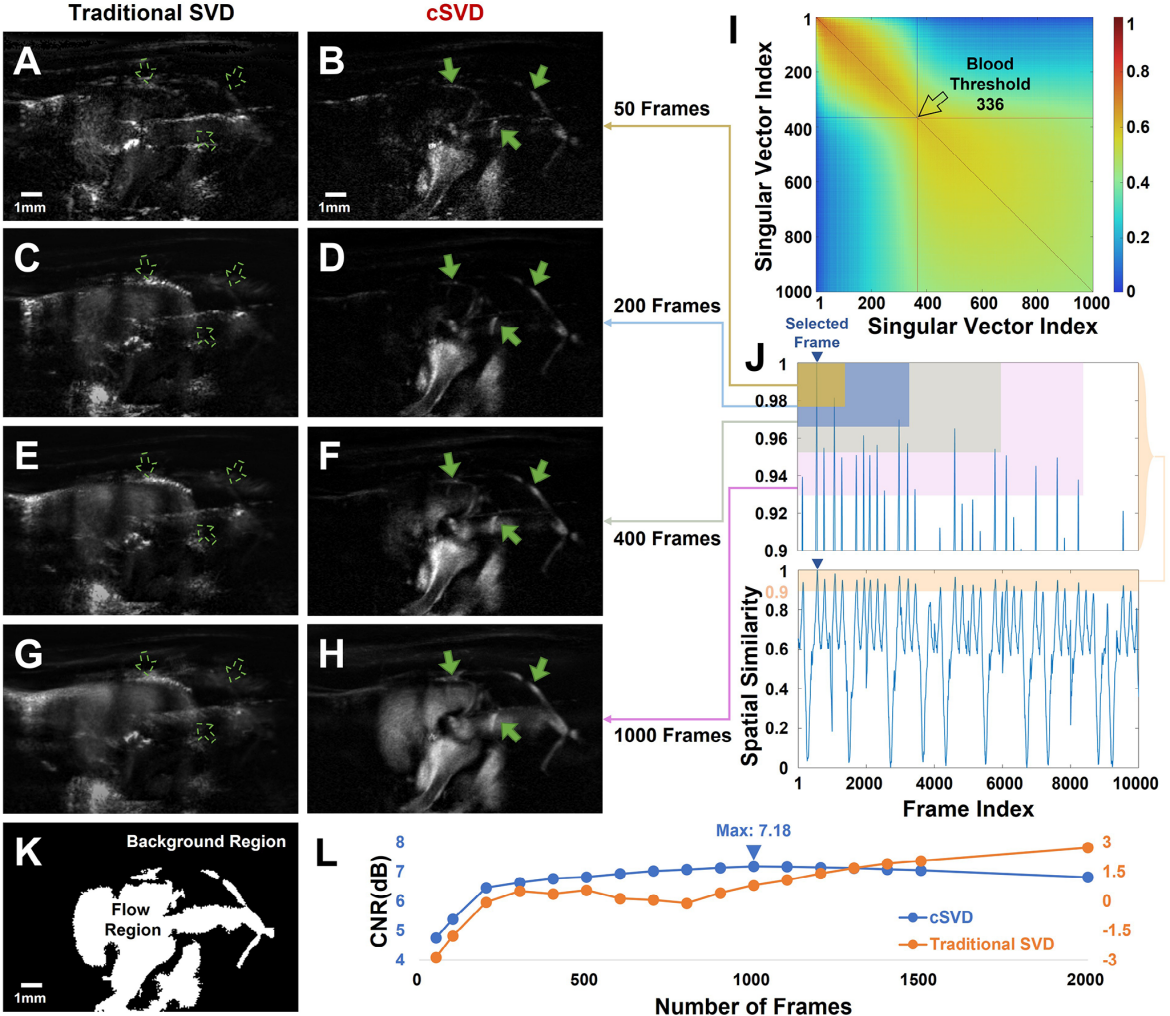
In vivo contrast‐free cardiovascular flow imaging results. A) Imaging results from sliding spatio‐temporal analysis of 50 adjacent frames; B) Imaging results from cSVD by selecting top‐50 highest‐similarity frames; C–H) Imaging results from sliding spatio‐temporal analysis and cSVD with corresponding parameters selected as 200, 400, and 1000, respectively; (I) Spatial similarity matrix of the spatial singular vector U obtained from the SVD on the top‐1000 highest‐similarity frames set; J) Spatial‐similarity sequence curve of the selected frames; K) Binary mask of (H); L) CNR curves of SVD and cSVD imaging results with different parameter selections (n = 3).

In this study, the selected frame was taken during the isovolumetric relaxation, and its spatial‐similarity sequence *R* was shown in the lower subplot of **Figure** **2**J, where the selected frame is indicated by a blue arrow. Normally, the interval between two adjacent peaks in *R* represents a cardiac cycle, while the interval between two adjacent valleys corresponds to a respiratory cycle due to the large deformations induced by respiration significantly reducing spatial‐similarity. The upper subplot of Figure 2J highlights *R* > 0.9, the top‐50 highest‐similarity frames set indicated by the yellow box were fed to SVD clutter filter to producing the cSVD imaging result shown in Figure 2B, while the flow imaging results shown in Figure 2D, F,H were processed via 200, 400, and 1000 highest‐similarity frames sets indicated by the blue, light green, and pink boxes, respectively. Figure 2A, C, E,G illustrate the imaging results obtained by the sliding spatio‐temporal analysis method in ref. [25], in which 50, 200, 400, and 1000 adjacent frames were directly fed to the SVD clutter filter. In the cSVD results, the regions indicated by solid green arrows represent coronary arteries, while in the sliding spatio‐temporal analysis results, dashed green arrows point to the corresponding locations.

Using the sliding spatio‐temporal analysis, the coronary arteries and intracardiac flow highlighted by the rightmost green arrow can be faintly observed in Figure 2A. However, as the number of frames increases, the imaging results become increasingly blurred. In contrast, cSVD significantly enhances the imaging capability of coronary arteries. As the number of high‐spatial‐similarity frame sets *M* increases, both intracardiac flow and coronary arteries become progressively clearer, with optimal clarity achieved at *M* is set to 1000. To quantitatively evaluate the impact of *M* on cSVD imaging results, adaptive threshold segmentation method^[^ ^37^ ^]^ was applied to Figure 2(H), generating a binary mask where the flow region appears white and the background region appears black. Based on Figure 2(H), contrast‐to‐noise ratio (CNR) was calculated using the formula referenced in [38]. The CNR curves of sliding spatio‐temporal analysis and cSVD were computed for frame number of 50, 100, 200, 300, 400, 500, 600, 700, 800, 900, 1000, 1100, 1200, 1300, 1400, 1500, and 2000. Consistent with empirical observations, the sliding spatio‐temporal analysis exhibited low CNR, whereas the cSVD imaging results demonstrated high CNR. When *M* was in the range of 800 to 1200, the difference in CNR was small, and the CNR reached its peak when *M* is 1000, also 10% of *n*_*t*_. Furthermore, the identical methodology was applied to calculate CNR curves of the ejection and filling phases (Figure S1, Supporting Information). These results consistently demonstrated that the optimal range for *M* occurs around 10% of *n*_*t*_. According to ref. [39], the spatial‐similarity of spatial singular vectors *U* can be used to determine the threshold for background suppression in SVD clutter filter. For the 1000 high‐similarity frames set corresponding to the selected frame, the spatial‐similarity matrix of U is shown in Figure 2I. After discarding the first 336 singular values and reconstructing, intensity averaging was performed to obtain the blood flow imaging result of the selected frame. The intracardiac and coronary blood flow fluctuations throughout the full cardiac cycle are provided in Section S1 (Supporting Information).

### 2.2 In Vivo Contrast‐Enhanced Coronary Arteries Blood Flow Imaging

**Figure 3:**
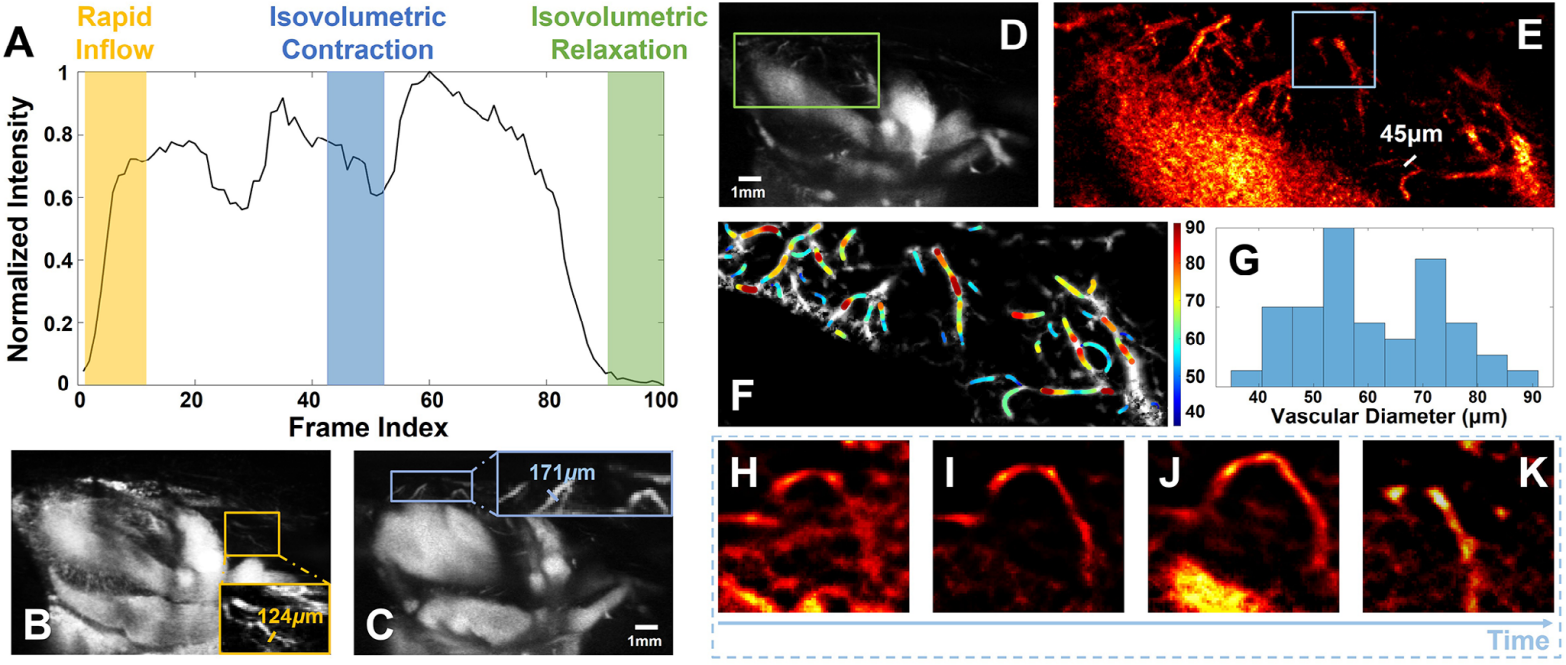
In vivo contrast‐enhanced cardiovascular flow imaging results. A). The normalized intensity fluctuation curve of cardiovascular flow fluctuation; B–D) Imaging result during rapid inflow phase, isovolumetric contraction phase and isovolumetric relaxation phase; E) UDAM imaging results of green ROI in (D); F) Vascular diameter measurement of (E); G) Vascular diameter distribution histogram of (E) (n = 3); H–K) Imaging results during different phase of blue ROI in (E).

**Figure** **3**A show the normalized intensity fluctuations over a full cardiac cycle. Figure 3B,C represents the imaging result during the rapid inflow phase and isovolumetric contraction phase. The ROI in Figure B,C are amplified and the cardiovascular marked by lines are measured by full‐width at half‐maximum (FWHM). As the optimal observation phase, the imaging results during isovolumetric relaxation phase shown in Figure 3D is reconstructed by UDAM (Figure 3E). After this SRUS strategy, the smallest measurable vascular diameter is approximately 45 µm. By counting the diameter distribution of cardiovascular in Figure 3F–G, we find that the diameters of numerous vessels are below the half of wavelength 54 µm. Besides, the Figure 3H,K show the UDAM imaging results of the coronary in the blue ROI of Figure 3E during different phase. In these results, we can observe the changes of the same vessel during a full cardiac cycle. The comparison of cSVD and dynamic UDAM imaging over a full cardiac cycle is provided in Section S2 (Supporting Information).

### 2.3 Dynamic Multi‐Slice 3D Coronary Arteries Blood Flow Imaging

**Figure 4:**
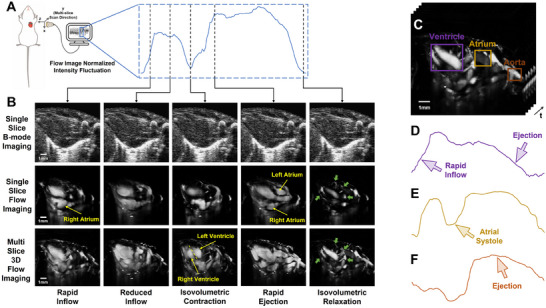
In vivo dynamic multi‐slice 3D contrast‐free cardiovascular flow imaging results. A) Schematic of ultrasound cardiac acquisition and the overall normalized intensity fluctuation curve of the dynamic multi‐slice 3D cardiovascular flow imaging result over the full cardiac cycle; B) Imaging results during rapid inflow phase, reduced inflow phase, isovolumetric contraction phase, rapid ejection phase, and isovolumetric relaxation phase; C) Dynamic multi‐slice 3D cardiac blood flow imaging result over the full cardiac cycle; D–F) Normalized intensity fluctuation curve of the blood flow corresponding to the purple, yellow, and brown ROIs in (C), representing ventricular, atrial, and aortic volume, respectively.

Similarly, 17 slices of newborn rat cardiac data were acquired and processed by cSVD. By temporal alignment and combination, the dynamic multi‐slice 3D coronary arteries blood flow imaging results over the full cardiac cycle was obtained. **Figure** **4**A shows the schematic of newborn rat cardiac data acquisition.

The curve on the right represents the normalized intensity fluctuations of the dynamic 3D coronary arteries blood flow imaging over a full cardiac cycle. Meanwhile, the five dashed lines correspond to the five columns below, which show the imaging results of a specific phase in the cardiac cycle. Accordingly, the imaging results in Figure 4B below include: the single‐slice B‐mode imaging of the newborn rat heart in the first row, the flow imaging of the same slice in the second row, and the dynamic 3D coronary arteries blood flow imaging visualized by Clear Volume in the third row.

The first column inFigure 4B represents imaging results during the rapid filling phase. In this phase, the blood flow from the atria rapidly into the ventricles. In the single‐slice flow imaging result, the region marked by yellow arrow corresponds to the right atrium. Besides, the second column represents the reduced filling phase, where the intracardiac flow continues to increase but at a slower rate. While the third column shows the isovolumetric contraction phase. In this stage, the ventricles continue to contract, but the total blood volume remains abiding while the intraventricular pressure increases. In the 3D flow imaging result, the boundaries between the two ventricles are clearly visible. Moreover, the fourth column represents the rapid ejection phase, where the blood from ventricles is ejected through the aorta, significantly reducing the intraventricular blood volume, and the left and right atria is indicated by yellow arrows. As described in Figure 2, the isovolumetric relaxation phase shown in last column is optimal phase for observing coronary arteries, and the region indicated by the green arrows highlight the typical coronary arteries.

Figure 4C illustrates the Dynamic multi‐slice 3D coronary arteries blood flow imaging results over the full cardiac cycle. The purple, yellow, and brown ROIs correspond to the ventricular, atrial, and aortic regions, respectively. Besides, the dynamic 3D coronary arteries blood flow imaging results and rotating 3D rendered flow imaging results during the isovolumetric relaxation phase are provided in Section S3 (Supporting Information). Figure 4D shows the ventricular volume fluctuations, where the volume rapidly increases during the filling phase and decreases to a minimum during the ejection phase; Figure 4E reflects the atrial volume fluctuations, where the volume decreases during atrial contraction phase, and then rapidly increases to a higher level and then stabilizes; Figure 4F corresponds to the aortic volume, which peaks during the ejection phase as the blood is ejected from the ventricles into the aorta, and remains low during other phases.

### 2.4 In Vivo Contrast‐Free Hepatic Flow Imaging

Also, to validate the applicability of cSVD to other rhythm beyond the fast‐paced cardiac rhythm, this study conducted ultrasound blood flow imaging experiments on the liver and intestines, which are influenced by both the fast‐paced cardiac rhythm and the slow‐paced respiratory rhythm. As an abdominal organ, the liver undergoes the complex flexible deformations influenced by cardiac rhythm, respiratory rhythm, and irregular intestinal peristalsis, posing a significant challenge for in vivo imaging.^[^ ^40^, ^41^, ^42^ ^]^ Furthermore, research on ultrasound blood flow imaging of the intestine remains an underexplored area, primarily due to the severe impact of motion artifacts caused by respiration and peristalsis.^[^ ^43^, ^44^ ^]^

**Figure 5:**
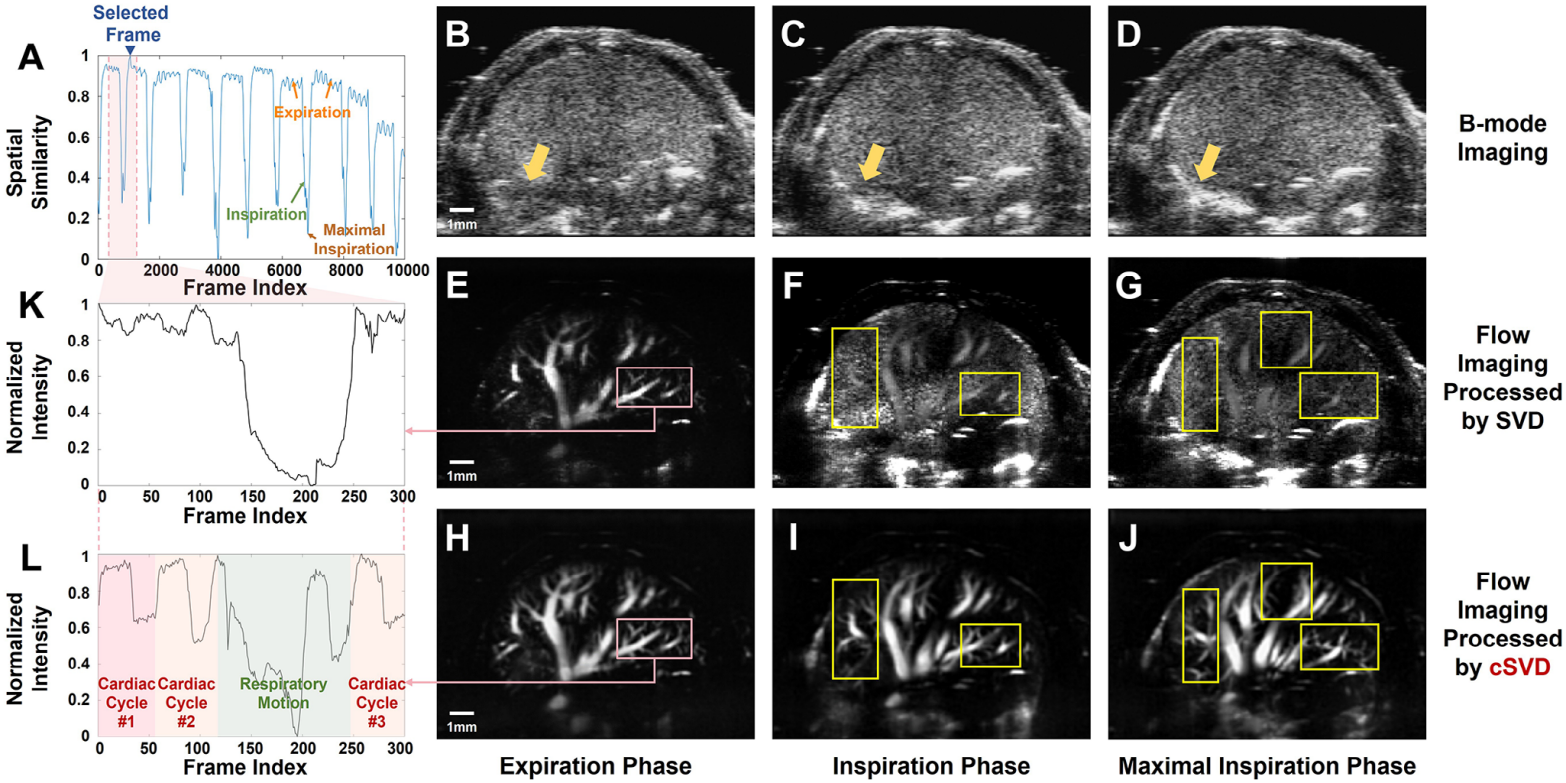
In vivo contrast‐free hepatic flow imaging results. A) Spatial similarity sequence of the selected frame; B–D) B‐mode imaging results during exhalation, inhalation, and maximum inhalation phases; E–G) Flow imaging results processed by SVD during the three phases; H–J) Flow imaging results processed by cSVD during the three phases; K) Normalized intensity fluctuations of the pink ROI in (E); L) Normalized intensity fluctuations of the pink ROI in (H).

**Figure** **5** provides a detailed analysis of hepatic imaging results. In this experiment, a frame during exhalation was chosen as the selected frame, and the spatial‐similarity sequence *R* is shown in Figure 5A. The selected frame is denoted by dark blue text and arrows, and the chosen respiratory cycle is indicated by pink dashed lines. Likewise, the high‐similarity frames are in the exhalation phase, and the interval between two adjacent peaks indicate a cardiac cycle. During inhalation, the similarity rapidly reduces due to the significant deformation of the liver, and the lowest similarity values generally correspond to the maximum inhalation phase. Besides, imaging results for these three states are shown in the three columns on the right.

Figure 5B–D show the B‐mode imaging results of the three different phases, with the golden arrows indicating regions where respiration induced liver motion; Figure 5E–G represent the blood flow imaging results obtained by applying SVD to 100 adjacent frames, discarding the first 20% of singular values and reconstructing; Figure 5H–J show the blood flow imaging results derived from cSVD. Compared to the imaging results processed by SVD, the ROIs marked by the yellow box suggest a significant improvement in imaging performance with cSVD, particularly in microvascular flow sensitivity. In addition, Figure 5K shows the intensity fluctuations within the pink ROI shown in Figure 5E during the selected respiratory cycle, which rapidly decreases during the inhalation and stabilizes during exhalation, without clearly revealing the flow fluctuations corresponding to the cardiac cycle.

Conversely, Figure 5L depicts the intensity fluctuations of the same ROI in Figure 5H, where significant variations flow volume are observed during each cardiac cycle, alongside larger fluctuations due to the respiratory motion. Furthermore, the cardiac cycles and time points influenced by respiratory motion are marked with red and green text, respectively.

In Section S4 (Supporting Information), the dynamic imaging results reveal the respiratory cycle of the liver, along with pronounced flow fluctuations during each cardiac cycle; while in Section S5 (Supporting Information), the ultrasound blood flow imaging results of the intestine are presented, filling a gap in previous research.

### 2.5 In Vivo Contrast‐Enhanced Renal Dynamic UDAM Imaging

**Figure 6:**
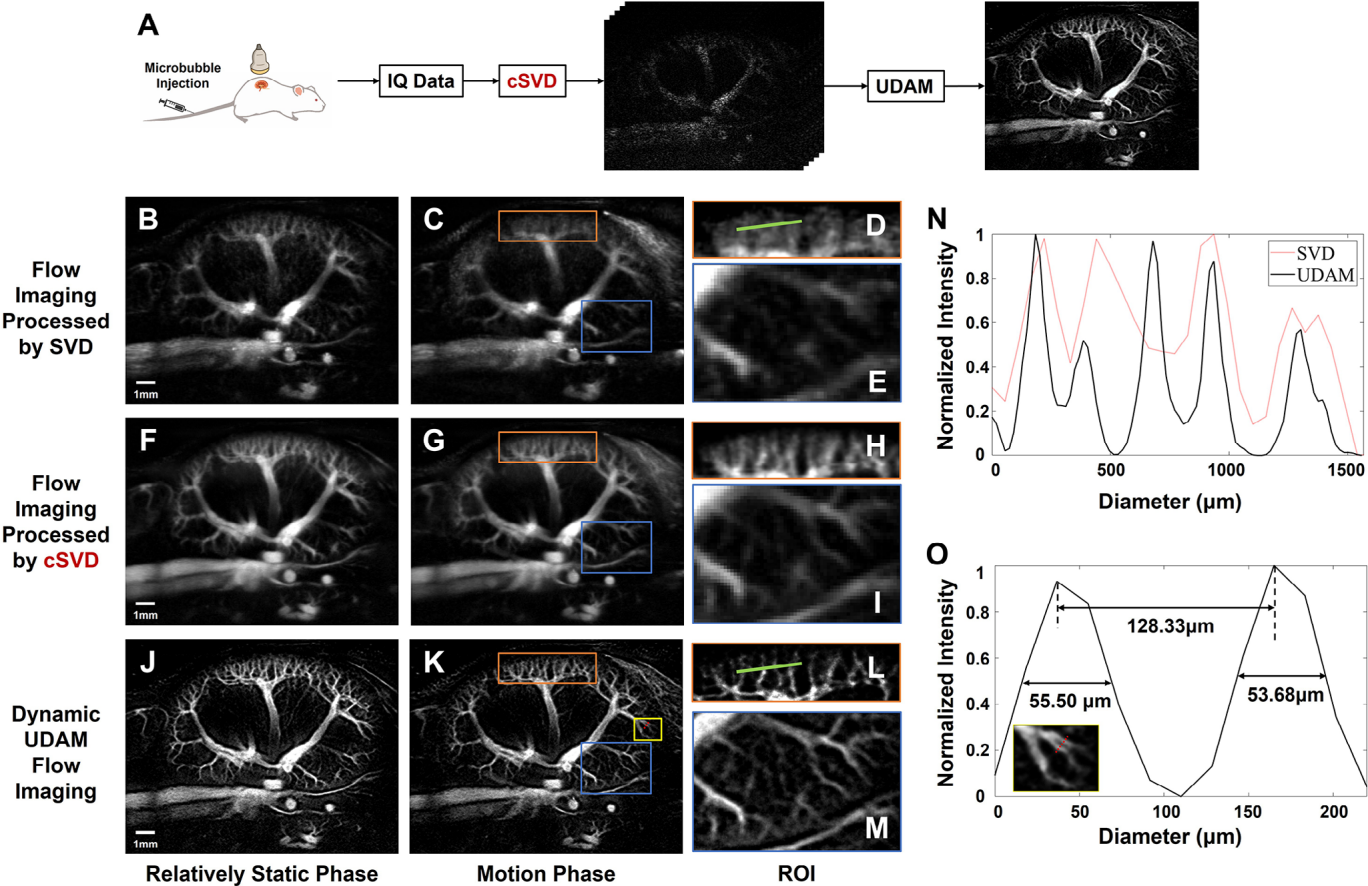
In vivo contrast‐enhanced renal dynamic UDAM imaging results. A) Schematic of renal dynamic UDAM framework; B–C) Flow imaging results processed by SVD; D–E) Corresponding ROIs marked in the same colors as in (C); F–I) Flow imaging results processed by cSVD; J–M) Corresponding dynamic UDAM imaging results processed by cSVD and UDAM; N) Comparison of vascular intensity distribution along the green line in subplots (D) and (L); O) The vascular intensity distribution along the red dashed line in the yellow‐bordered ROI in (K), and the vessel diameter measured by FWHM.

Further, **Figure** **6** verifies the dynamic UDAM on the renal flow imaging across the full respiratory cycle. In this work, the selected frames were from two distinct phases: relatively static phase which is unaffected by respiration, and motion phase due to respiratory motion. The respective ultrasound blood flow imaging results are shown in the first and second columns.

Figure 6B,C shows the blood flow imaging results processed by SVD, while Figure 6D,E displays the corresponding ROIs with the same color in Figure 6C. Similarly, Figure 6F–I represent the results processed by cSVD, and Figure 6J–M show the dynamic UDAM blood flow imaging results achieved by combining cSVD with the UDAM strategy. In the dynamic UDAM imaging results, the blood flow sensitivity, particularly for cortical microvascular flow sensitivity, is significantly improved. The SVD‐based imaging results remain comparable in the relatively static phase, however in the motion state, the cortical microvascular details become blurred. In contrast, the cSVD and the dynamic UDAM imaging results maintain high blood flow sensitivity even in the motion phase. Figure 6N illustrates the vascular intensity distribution along the green line in Figure 6D,L. Obviously, the SVD‐based imaging results fail to identify the microvascular in the renal cortex, while the dynamic UDAM imaging strategy is capable of imaging and distinguishing the interlobular vascular. Besides, Figure 6O shows the vascular intensity distribution along the red dashed line within the yellow‐bordered ROI in Figure 6K. Using FWHM measurements, the dynamic UDAM imaging strategy can detect microvascular with a diameter of 53.68 µm in the motion state and clearly distinguish two vessels separated by 128.33 µm. Especially, the renal motion following the respiratory rhythm in this slice, with a comparison across SVD, cSVD, and dynamic UDAM imaging results, can be observed in Section S6 (Supporting Information).

## 3 Discussion

Coronary arteries blood flow imaging has long been a great challenge in ultrasound flow imaging, as the vigorous motion of myocardial tissue induces frequency aliasing in conventional SVD‐based clutter filters,^[^ ^23^, ^28^, ^36^, ^45^ ^]^ thus leading to a significant reduction in imaging quality.^[^ ^46^ ^]^ In recent years, with the development of ultrafast ultrasound imaging strategy^[^ ^20^, ^21^, ^22^, ^23^ ^]^ that ensure high‐frame‐rate, several cardiovascular flow imaging techniques have been proposed.^[^ ^5^, ^24^, ^25^, ^26^ ^]^ However, these technologies can only use during diastole only as well.

Whereas for ultrasound coronary arteries blood flow imaging, the capability to imaging throughout the full cardiac cycle is of great significance, for that could provide more dimensions and supporting evidence for assessing the coronary arteries diseases while that cannot be achieved by imaging only during diastole.^[^ ^24^ ^]^ For instance, the unique flow information contained in the blood flow imaging results from all phases within the cardiac cycle shown in Figure 4. Unfortunately, according to our research, there has been no study on dynamic coronary arteries blood flow imaging of the full cardiac cycle, either of animals or humans.

The proposed cSVD in this study is a spatial‐similarity‐clustering‐based SVD method, which reconstructs the blood flow imaging using the inherent spatio‐temporal information provided by high‐similarity frames, allowing to capture blood flow fluctuations during the full cardiac cycle, leading to significantly improves the image quality and resolution of ultrasound blood flow imaging. Reviewing our past experiments, we found that there is a periodicity in the spatial‐similarity sequence *R*, and we could use the periodic feature to cluster the high‐similarity frames from phase of multi‐cycle, then reconstruct the ultrasound blood flow imaging effectively.

There is a practical application of the above ideas in MRI,^[^ ^27^, ^30^, ^31^, ^32^, ^33^, ^34^, ^35^ ^]^ but unlike the respiratory gating acquisition strategy of MRI, we use the information contained in B‐mode imaging for motion measurement instead of the additional ECG. Furthermore, the high‐frame‐rate ultrasound imaging strategy‐based cSVD does not require the lengthy acquisition times compare to MRI. Only a few dozen seconds of acquisition time are sufficient for reconstructing the dynamic blood flow imaging results.

The experiment of animals validates the superior imaging capability of cSVD for organs influenced by respiratory rhythms through in vivo ultrasound blood flow imaging of the kidney, liver and intestines (in Section S4– S6, Supporting Information), demonstrating the cSVD strategy is not only applicable for fast‐paced cardiac rhythms, but also for relatively slow‐paced respiratory rhythms. More specifically, the in vivo organs in experiments correspond to different motion patterns: the cardiac imaging illustrates vigorous and rapid contraction and expansion deformations, the hepatic imaging presents complex flexible deformations, the intestinal imaging shows peristalsis deformations, and the kidneys have both translation and rotation deformations, all of which demonstrate that cSVD can be applied to various motion patterns in different organs.

Notably, the proposed cSVD in this study is not limited to contrast‐free imaging. In contrast‐enhanced imaging, the acoustic response of contrast agents is much stronger than that of erythrocytes, which results in a significant improvement in imaging quality and blood flow sensitivity.^[^ ^47^, ^48^, ^49^, ^50^ ^]^ Additionally, with the addition of contrast agents, the spatial‐similarity become more reliable, ensuring that the high‐similarity frame sets remain quasi‐static, thus allowing cSVD to filter out clearer blood flow. Further, as the contrast agent filling the microvascular, the blood flow sensitivity of microvascular, such as the renal cortex flow, would improve significantly. More importantly, in contrast‐enhanced imaging, cSVD can be combined with the UDAM strategy.^[^ ^36^ ^]^

The UDAM is a SRUS technique that overcomes the acoustic diffraction limit based on radial fluctuations, which is suitable for super‐resolution imaging under moderate to high contrast agent concentrations and can reconstruct SRUS imaging results via only a few hundred frames of images. Unlike the ULM,^[^ ^51^, ^52^, ^53^, ^54^ ^]^ the UDAM does not rely on the tracking of microbubbles to accumulate their trajectories and achieve SRUS imaging, thus does not require the image sequence to be continuous in the slow‐time direction, which perfectly matches the quasi‐static high‐similarity frame sets clustered by cSVD are not continuous in the slow‐time direction. As a result, the UDAM can provide more detailed vascular structures and significantly improve image resolution, offering a feasible approach for in vivo SRUS imaging under free‐breathing conditions. As for our experiment, the SRUS cardiovascular and renal imaging via UDAM showed effectiveness. Clearly, UDAM also has significant potential for dynamic blood flow imaging.

In addition, our proposed cSVD method was validated on the Verasonics Vantage 256 high‐frequency system and the Vevo F2 system using ultrafast plane wave and focused wave imaging strategies correspondingly, which demonstrates the compatibility of cSVD with the different devices and imaging strategies. It must be mentioned that proposed cSVD does require a certain level of image quality, when the image quality is unsatisfactory, the calculation of spatial‐similarity is meaningless and the high‐similarity frame sets do not remain quasi‐static. Moreover, due to the cSVD relies on finding valid spatio‐temporal information from total acquired data based on spatial‐similarity, in cases where no similar frames are available, such as when the transducer unexpectedly shifts or when abnormal behavior of the experimental animal leads to unexpected changes in the acquired imaging regions, either cSVD and previous image registration‐based approaches are ineffective.

Additionally, the *M* is an important parameter that can be determined by plotting the spatial‐similarity sequence *R*, based on visual assessment and quantitative CNR analysis in Figure 2L, the optimal selection range for parameter *M* was identified to be around 10% of *n*_*t*_. Besides, taking into acquisition time and computational costs, *n*_*t*_ ranging between 10,000 and 30,000 is sufficient for dynamic coronary arteries blood flow imaging.

Last but not least, although the current cSVD is an offline post‐processing method, it can certainly be integrated into the acquisition hardware in practical applications. By performing a few seconds of warming‐up pre‐acquisition to obtain the ultrasound echo IQ signals and then computing spatial‐similarity, clustering, SVD clutter filtering, and UDAM reconstructing on the current acquired frame, real‐time dynamic UDAM flow imaging results can be obtained.

## 4 Conclusion

As an innovative post‐processing method, the proposed cSVD is designed for dynamic ultrasound coronary arteries blood flow imaging, which reconstructs the blood flow imaging results via the inherent spatio‐temporal information of high‐similarity frames from multi‐cycle, and enables the display of the blood flow fluctuations of heart during full cardiac cycles.

Experiments in animals demonstrate the outstanding performance for motion artifacts suppression and imaging quality improvement in cSVD‐based ultrasound coronary arteries blood flow imaging under free‐breathing conditions, from which we believe that cSVD would provide a novel and powerful approach for in vivo ultrasound imaging of human organs such as heart, liver, and kidney under free‐breathing conditions as well.

cSVD has clear significance for the high‐frame‐rate clinical ultrasound devices and offers a feasible solution for ultrasound blood flow imaging of motive organs, especially coronary arteries blood flow imaging and real‐time diagnosis. Further, combining with UDAM, cSVD offers a novel path for dynamic SRUS imaging.

## 5 Experimental Section

### Ultrasound Imaging System

The Verasonics Vantage 256 high‐frequency system (Verasonics Inc., Kirkland, WA) combined with an L22‐8 high‐frequency linear array probe (Vermon SA, Tours, France) with a center frequency of 15 MHz and a bandwidth of 8‐22 MHz. The imaging strategy used was a common seven‐angle coherent compounding ultrafast plane‐wave imaging technique, with pulse‐repetition frequency (PRF) of 25 kHz and compounding frame rate of 500 Hz. Additionally, the Vevo F2 system (VisualSonics Inc., Toronto, Canada) was used in conjunction with an UHF46x probe, with a center frequency of 30 MHz and a bandwidth of 20–46 MHz. The imaging strategy used the default focused wave imaging strategy in cardiac mode with a compounding frame rate of 600 Hz.

### Animal Studies

All experimental procedures were reviewed and approved by the local animal care committee of Peking University (AAIS‐ZhangJ‐9). This study involved three male BALB/c mice (Beijing Vital River Laboratory Animal Technology; 8–10 weeks old; 20–25 g) and three Sprague‐Dawley newborn rats (Beijing Vital River Laboratory Animal Technology; 3 days old; 8–14 g) for ultrasound data acquisition. All the animals were kept under standard laboratory conditions (temperature ±20 °C, 12‐h light / 12‐h dark) and were allowed free access to water and food.

### Data Acquisition of Mice

The data of mice was acquired by the Verasonics Vantage 256 high‐frequency system and the L22‐8 high‐frequency linear array probe. Before imaging, the chest and dorsal hair of the mice were removed with clippers and depilatory cream. The mice were placed in a supine position on the operating table and was anesthetized with isoflurane gas (3.0% for induction, 1.0% for maintenance). The probe was aligned along the cardiac long axis to acquire 10,000 frames of single‐slice cardiac data in contrast‐free mode. After contrast‐free acquisition, a 0.05 mL of long‐lasting microbubble SuperVue‐MB solution (Nanjing Leapsonics Medical Technology Co., Ltd., China) with a concentration of 1 × 10^8^ mL was injected into mice through the tail vein. Subsequently, the L22‐8 probe was used to acquire 10,000 frames of single‐slice cardiac data in contrast‐enhanced mode. Next, the mice were placed in a prone position and the probe was aligned along the renal long axis for acquisition of 10,000 frames of single‐slice renal data in contrast‐enhanced imaging mode.

### Data Acquisition of Newborn Rats

The data of mice was acquired by the Vevo F2 system and the UHF46x probe. The newborn rats were placed in a supine position on the operating table and was anesthetized with isoflurane gas (3.0% for induction, 1.0% for maintenance). For the dynamic multi‐slice 3D coronary arteries blood flow flow imaging, a custom probe holder was used to fix the UHF46x probe along cardiac long‐axis, while allowing precise movement of 200 µm per step along the short‐axis direction under a stepper motor. For each newborn rat, 17 slices were acquired, and each slice contained 10,000 frames of beamformed RF data. Subsequently, the probe was oriented perpendicular to the longitudinal axis. The single‐slice hepatic and intestinal beamformed RF data was acquired by the same imaging setting.

### cSVD

As shown in Figure 1, the RF data of *n*_*t*_ frames acquired were demodulated to obtain IQ data *S*(*x*, *z*, *t*), and the magnitude of the complex IQ data was computed to obtain the ultrasound B‐mode images,

(1) C(x,z,t)=|S(x,z,t)|

where *x* represents the lateral dimension (along the transducer array), *z* represents the depth dimension of the ultrasound probe, *t* represents the time dimension, and the size of *S* are *n*_*x*_, *n*_*z*_, *n*_*t*_.

Given the *i*‐th frame of the B‐mode image *C*(*x*, *z*, *i*), the spatial‐similarity sequence between it and each frame in *C*(*x*, *z*, *t*) was derived,

(2) $$R_{j}^{i}=Corr\left((C(i),C(j)\right),j=1,2,\text{…},n_{t}$$

The quasi‐static high‐similarity frames set *S*(*x*, *z*, {*K*_*i*_}), formed by concatenating the IQ data corresponding to the indices {*K*_*i*_} of the top‐*M* highest‐similarity frames, was processed through an adaptive SVD clutter filter based on the spatial singular vector similarity.^[^ ^39^ ^]^ Then, the *i*‐th frame flow imaging result *F*(*x*, *z*, *i*) was obtained by performing intensity averaging (Average Intensity, AVG) on the magnitude of the filtered complex spatio‐temporal matrix *S*′(*x*, *z*, {*K*_*i*_}),

(3) $$F\left(x,z,i\right)=\frac{\sum_{k}^{M}S^{\prime }\left(x,z,k\right)}{M},k\in \left\{K_{i}\right\}$$

Subsequently, processing each frame in a full motion cycle and concatenating the results, dynamic ultrasound flow imaging results was obtained.

In all experiments, 10,000 frames were acquired per slice, and *M* was set to 1000, which is 10% of *n*_*t*_, and the SVD threshold was determined by adaptive method.^[^ ^39^ ^]^ Typically, to reduce the computational cost, the study sampled the full motion cycle at even intervals along slow‐time direction. For cardiac imaging, 100 frames were downsampled from the full motion cycle, and cSVD reconstruction was applied to these frames to produce the dynamic blood flow imaging results. For hepatic, intestinal, and renal dynamic blood flow imaging, 300, 300, and 280 frames were downsampled, respectively.

### UDAM

For contrast‐enhanced imaging, this study used the SRUS strategy UDAM^[^ ^36^ ^]^ to further improve image resolution. UDAM was a super‐resolution strategy based on radial fluctuations that overcomes the acoustic diffraction limit, which was derived from the optical super‐resolution method Super‐resolution Radial Fluctuations (SRRF).^[^ ^55^ ^]^ In this study, the NanoJ‐SRRF package in ImageJ was used to perform UDAM reconstruction on *S*′(*x*, *z*, {*K*_*i*_}). Every frame of *S*′(*x*, *z*, {*K*_*i*_}) was processed via UDAM reconstruction based on the Temporal Radiality Maximum (TRM) method. The standard deviation (STD) of the reconstructed image stack was then calculated to obtain the final UDAM reconstruction results. In all experiments, the same parameters were used (Ring Radius, 0.5; Radiality Magnification, 3; Axes in the Ring, 3; other parameters were set to default).

### Data and Statistical Analysis

All acquired data were exported and post‐processed using Matlab 2022b (MathWorks Inc., MA, USA) and ImageJ (version 1.54f; National Institutes of Health, Bethesda, MD). Each single‐slice images was processed through “Subtract Background” (Rolling ball radius, 200 pixels) and adjusted to the same dynamic range via ImageJ. For the contrast‐free coronary arteries blood flow imaging, the CNR was used to quantify the imaging performance of cSVD and traditional SVD with different parameter selections (n = 3), and the CNR curves of cSVD during different phases were shown in Figure S1 (Supporting Information) (n = 3). For the contrast‐enhanced coronary arteries blood flow imaging, the UDAM results were filtered by Wiener filter^[^ ^56^ ^]^ and Frangi filter^[^ ^57^ ^]^ to derive binary mask and skeleton of vascular, while the distribution of vascular diameters was quantified through histogram analysis (n = 3). For dynamic multi‐slice 3D coronary arteries blood flow imaging, different slices were phase aligned and combined to 4D data (*x*, *y*, *z*, *t*), after ‘Mean 3D’ (X radius, 1; Y radius, 3; Z radius, 1) and resize to the same sizes in each direction, visualization the 4D imaging and the rotating 3D imaging was rendered by Clear Volume package^[^ ^58^ ^]^ in ImageJ.

## Conflict of Interest

The authors declare no conflict of interest.

## Supporting information

Supporting Information

Supplemental Video 1

Supplemental Video 2

Supplemental Video 3

Supplemental Video 4

Supplemental Video 5

Supplemental Video 6

## Data Availability

Research data are not shared.
