# Supplementary material for: In Vivo Dynamic Coronary Arteries Blood Flow Imaging Based on Multi‐Cycle Phase Clustering Ultrafast Ultrasound
Source: Adv Sci (Weinh). 2025 Jun 30;12(31):e05485. doi: 10.1002/advs.202505485 (PMC12376710; doi:10.1002/advs.202505485)
Supplement: Supplementary file 1 — Supporting Information [file ADVS-12-e05485-s005.docx]

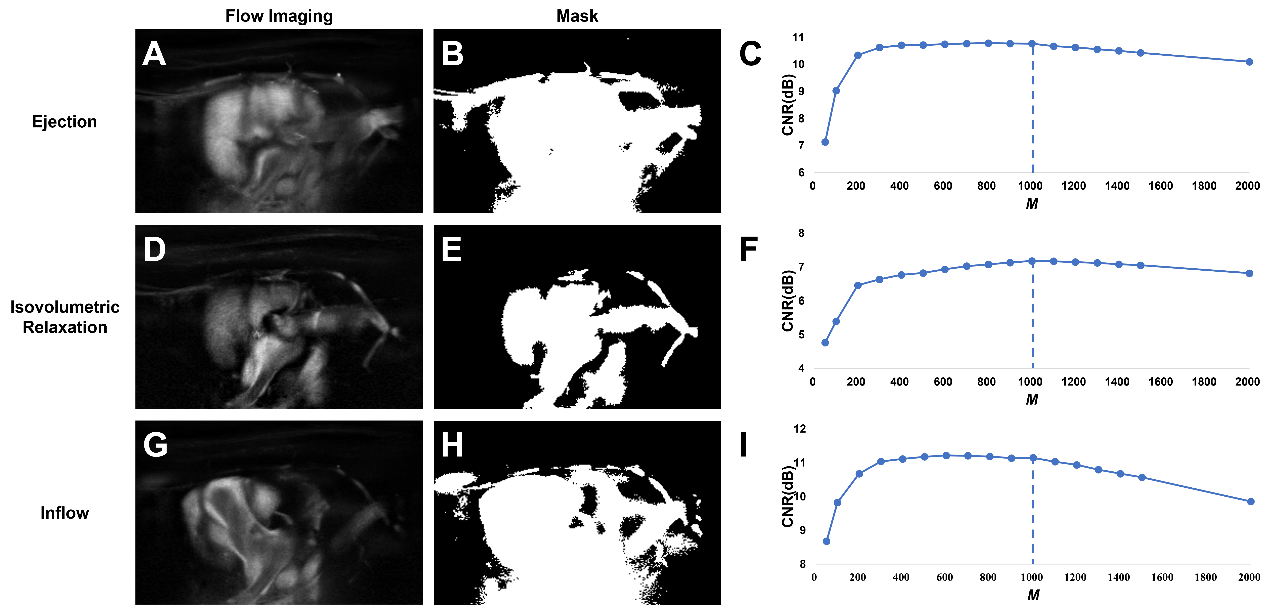


Figure S1. CNR curves of cSVD imaging results with different $M$ during different phases (n = 3). The three rows from top to bottom are CNR curves during the ejection phase, isovolumetric relaxation phase, and filling phase. All three results confirm that $M=10\%\times n_{t}$ is an appropriate parameter choice.
